# Diastolic function is a strong predictor of mortality in patients with chronic kidney disease

**DOI:** 10.1186/1471-2369-14-280

**Published:** 2013-12-23

**Authors:** Ahmad Farshid, Rajeev Pathak, Bruce Shadbolt, Leonard Arnolda, Girish Talaulikar

**Affiliations:** 1 Cardiology Unit, The Canberra Hospital, PO Box 11, Woden, ACT 2605, Australia; 2 Centre for Advances in Epidemiology and Information Technology, The Canberra Hospital, Canberra, Australia; 3 Renal Unit, The Canberra Hospital, Canberra, Australia; 4 Australian National University, Canberra, Australia

**Keywords:** Chronic kidney disease, Echocardiography, Diastolic function, Cardiovascular disease, Troponin T

## Abstract

**Background:**

Cardiovascular disease is a major cause of death in patients with stage 4–5 Chronic Kidney disease (CKD, eGFR < 30). There are only limited data on the risk factors predicting these complications in CKD patients. Our aim was to determine the role of clinical and echocardiographic parameters in predicting mortality and cardiovascular complications in CKD patients.

**Methods:**

We conducted a prospective observational cohort study of 153 CKD patients between 2007 and 2009. All patients underwent echocardiography at baseline and were followed for a mean of 2.6 years using regular clinic visits and review of files and hospital presentations to record the incidence of cardiovascular events and death.

**Results:**

Of 153 patients enrolled, 57 (37%) were on dialysis and 45 (78%) of these patients were on haemodialysis. An enlarged LV was present in 32% of patients and in 22% the LVEF was below 55%. LV mass index was increased in 75% of patients. Some degree of diastolic dysfunction was present in 85% of patients and 35% had grade 2 or higher diastolic dysfunction. During follow up 41 patients (27%) died, 15 (39%) from cardiovascular causes. Mortality was 24.0% in the non-dialysis patients versus 31.6% in patients on dialysis (p=ns). On multivariate analysis age >75 years, previous history of MI, diastolic dysfunction and detectable serum troponin T were significant independent predictor of mortality (P < 0.01).

**Conclusion:**

Patients with stage 4–5 CKD had a mortality rate of 27% over a mean follow up of 2.6 years. Age >75 years, history of MI, diastolic dysfunction and troponin T were independent predictors of mortality.

## Background

Cardiovascular disease is the major cause of death in patients with advanced chronic kidney disease (CKD stages 4–5, eGFR <30 mL/min/1.73 m^2^), accounting for about 40% of deaths in international registries [1]. The risk of cardiovascular mortality is more than 10 times higher in this population compared with an age, sex, and race matched population [2]. Data on cardiovascular risk factors from the general population cannot simply be extrapolated to CKD patients as they are subject not only to traditional risk factors, but also to CKD-related risk factors such as inflammation, increased calcium and phosphorus products, uremic toxins, anemia, and fluid overload [3,4]. Additionally, CKD patients show a very high prevalence of vascular and valvular calcification which have been shown to be associated with increased arterial stiffness and adverse outcomes [5,6].

Echocardiography provides a non-invasive assessment of cardiac structures and function. There is limited data on echocardiographic parameters predicting cardiovascular complications in patients with advanced CKD, including those who have not commenced dialysis [7-9]. Our aim was to assess the role of echocardiographic and clinical parameters and cardiac biomarkers in predicting death and cardiovascular complications in patients with advanced CKD and to compare non-dialysis patients with those on dialysis.

## Methods

### Study subjects

We conducted a 4 year prospective observational study, enrolling 153 consecutive patients between May 2007 and May 2009. The setting of the study was the Renal Outpatient Clinic of a Public tertiary referral hospital with approximately 400 outpatients with GFR <30 ml/min and 200 dialysis patients. Consecutive adult patients with stage 4–5 CKD (eGRF < 30 mL/min/1.73 m^2^ using the modified MDRD equation) attending the Clinic were screened and invited to take part, including patients who were already on dialysis. Patients were excluded if they were unable or unwilling to return for regular follow up or were at high risk of death due to any cause within 6 months of enrolment. The investigation conforms with the principles outlined in the Declaration of Helsinki and was approved by the Australian Capital Territory Human Research Ethics Committee. All subjects gave informed written consent to take part in the study. Demographic and clinical characteristics were recorded and patients underwent echocardiography and blood tests (full blood count, electrolytes, urea and creatinine, calcium, phosphate and serum troponin T) at the time of entry into the study.

### Troponin T analysis

Analysis was carried out on the Roche Elecsys1010 (Roche Diagnostics Australia, Sydney, Australia). The limit of detection for this assay is 0.01 mcg/L with assay coefficients of variation (CV) being 6.0% at 0.11 mg/L and 2.5% at 2.5 mg/L.

### Echocardiography

2D and Doppler echocardiography was carried out on all patients lying in the left decubitus position at baseline. All studies were performed by a single experienced sonographer blinded to the clinical details of patients using a Philips iE33 echocardiographic system with a S5-1 probe. For patients on dialysis all studies were performed within 24 hour after dialysis. All echocardiographic data were measured according to the guidelines of the American Society of Echocardiography (ASE) [10]. LV mass was calculated using the ASE-recommended formula for estimation of LV mass from linear dimensions and was indexed to body mass. The left ventricular ejection fraction (LVEF) was measured in the apical views using Simpson’s method. Diastolic function was assessed using several parameters including the pattern of mitral inflow and the ratio of peak early (E) filling velocity to late diastolic filing (A) velocity (E/A ratio), deceleration time of early filling velocity (DT), and the isovolumic relaxation time (IVRT). PW Tissue Doppler Imaging was performed in the apical views to acquire mitral annular velocities. The ratio of early mitral inflow velocity to Tissue Doppler velocity e’ (E/e’) was used for the estimation of LV filling pressures. Diastolic function was classified into grades I-IV according to the ASE guidelines [11].

### Endpoints and Follow up

The primary endpoint was death from any cause. The secondary endpoint was the incidence of Major Adverse Cardiovascular Events (MACE) including death, myocardial infarction (MI), unstable angina, stroke and heart failure. MI was defined according to ESC guidelines 2007 as a rise and fall of troponin-I with at least one value above the 99^th^ percentile of the upper reference limit together with evidence of myocardial ischaemia [12]. Unstable angina was defined as cardiac ischaemic pain associated with ≥ 1 mm ST segment depression on ECG resulting in hospitalisation. Cardiac failure was defined according to the ESC definition of heart failure (2005) as presentation to a health care facility with symptoms of heart failure (typically breathlessness or fatigue), or ankle swelling and objective evidence of cardiac dysfunction at rest [13]. Cerebrovascular accident was defined as in the TREAT Study as a focal neurological deficit resulting from a vascular cause involving the central nervous system with a sudden onset and lasting longer than 24 hours [14]. Follow up of the patients was by clinic visits, phone calls and review of the clinical notes every six months.

### Statistical analysis

In addition to descriptive analyses, Cox proportional hazards models were used to test the univariate and multivariate associations between survival and the subjects’ risk factors. In the multivariate analysis, after adjusting for age and gender as well as diabetes and other risk factors (hypertension, smoking, hyperlipidaemia), a likelihood ratio method for factor inclusion was employed. Hazard ratios and the associated 95% confidence intervals and p values are shown in the results. SPSS version 19.0 was used to conduct the analyses and a two sided p value of <0.05 was considered significant.

## Results

Baseline characteristics for 153 enrolled patients are shown in Table 1. Patients on dialysis made up 38% of the study population, 78% of whom were on haemodialysis. A history of MI was present in 20% of patients and 46% were diabetic. An enlarged left ventricle was present in 32% and in 22% the LVEF was below 55%. LV mass index was increased in 75% of patients. Some degree of diastolic dysfunction was present in 85% of patients and 35% had grade 2 or higher diastolic dysfunction. Diastolic function could not be assessed in 16 patients (10%), mostly due to presence of atrial fibrillation. Follow up was for a mean of 2.6 years (median 2.89 years) and was available in all but 1 patient who moved to another state.

**Table 1:** Baseline characteristics of patients

	**Number (%)**
Mean age (year)	65.6 ± 14.0
Male gender	88 (57.5%)
**Medical history**	
Hypertension	127 (85%)
Diabetes	69 (46%)
Oral hypoglycaemics	25 (17%)
Insulin	34 (23%)
Heart failure	20 (13%)
Stroke	23 (15%)
Hyperlipidaemia	90 (60%)
Current smoker	15 (10%)
Atrial fibrillation	10 (6.5%)
AMI	30 (20%)
PCI	25 (16%)
CABG	17 (11%)
Patients on dialysis	56 (37%)
Haemodialysis	45 (29%)
Peritoneal dialysis	13 (8%)
**Aetiology of kidney disease:**	
Diabetes	48 (31%)
Glomerulonephritis	39 (25%)
Vascular	13 (8%)
Hypertension	10 (7%)
Vesicoureteric reflux	9 (6%)
Congenital	6 (4%)
Other	28 (18%)
**Medications**	
ACEI/ARB	86 (56%)
Diuretic	58 (38%)
Beta blocker	64 (42%)
Statin	92 (60%)
Calcium antagonist	60 (39%)
Aspirin	42 (27%)
EPO	65 (42%)

Clinical, biochemical and echocardiographic results as well as incidence of adverse events for dialysis and non-dialysis patients are shown in Table 2. There was a higher incidence of LV dysfunction and troponin T elevation in dialysis patients but there were no other significant differences between the two groups. Mortality was 24% in the non-dialysis patients versus 31.6% in patients on dialysis (p=ns).

**Table 2:** Clinical and echocardiographic characteristics in dialysis and non-dialysis patients

	**Non-dialysis**	**Dialysis**	**P value**
Number	96	57	NA
Mean age	69.7 ± 11.7	58.7 ± 15.0	<0.0001
Female sex	40 (42%)	25 (44%)	NS
Diabetes	45 (47%)	24 (42%)	NS
History of MI	18 (19%)	12 (21%)	NS
**Blood indices:**			
Haemoglobin (g/L)	123.8 ± 15.4	117.0 ± 12.3	0.0058
White cell count	7.38 ± 2.0	7.57 ± 2.13	NS
Platelet count	238 ± 63	232 ± 88	NS
Sodium	139.9 ± 2.6	137.4±	<0.0001
Potassium	4.46 ± 0.59	4.41±	NS
Urea (mmol/L)	18.9 ± 6.9	19.2 ± 6.7	NS
Creatinine (umol/L)	255 ± 88	646 ± 229	<0.0001
eGFR (mL/min/1.73 m^2^)	22.3 ± 7.4	8.3 ± 4.9	<0.0001
Calcium (mmol/L)	2.40 ± 0.12	2.38 ± 0.17	NS
Phosphate (mmol/L)	1.31 ± 0.29	1.84 ± 0.55	<0.0001
Parathyroid hormane	23.6 ± 29.1	51.4 ± 48.6	<0.0001
Troponin T detected	36 (38%)	44 (79%)	<0.001
**Echocardiographic results**			
LV diameter (mm)	49.5 ± 5.9	49.8 ± 6.2	NS
LV ejection fraction	62.1 ± 10.0	61.0 ± 14.0	NS
LV mass index	121.7 ± 35.4	126.8 ± 33.6	NS
Left atrial area (cm^2^)	22.1 ± 6.0	21.6 ± 4.9	NS
Aortic peak velocity (m/s)	1.50 ± 0.42	1.57 ± 0.47	NS
Mitral regurgitation (moderate or severe)	8 (8.3%)	6 (10.5%)	NS
Tricuspid regurgitation peak velocity (m/s)	2.53 ± 0.44	2.59 ± 0.45	NS
**Diastolic grade:**			
Grade 0	14 (16.3%)	7 (13.7%)	NS
Grade 1	48 (55.8%)	24 (47.1%)	NS
Grade 2	16 (18.6%)	15 (29.4%)	NS
Grade 3-4	8 (9.3%)	5 (9.8%)	NS
Indeterminate grade	9 (9.5%)	7 (12.1%)	NS
**Clinical outcomes**			
Death	23 (24.0%)	18 (31.6%)	NS
MACE	27 (28.1%)	24 (42.1%)	0.06

*LVEF*, Left ventricular ejection fraction; *LVMI*, Left ventricular mass index; *MACE*, Major adverse cardiovascular events, diastolic function was classified into grades 1–4 according to the ASE guidelines.

Adverse events during follow up for the entire cohort are shown in Table 3. During this time 41 patients (27%) died, 15 (39%) of whom died from cardiovascular causes. Survival analysis was performed to determine univariate clinical and echocardiographic factors predictive of death and adverse cardiovascular events (Table 4). Clinical predictors of death included age >75 years and a history of MI. Univariate echocardiographic predictors of mortality included enlargement of left ventricle and the left atrium, low LVEF, increased LV mass index and grade 2 or higher diastolic dysfunction. On multivariate analysis (Cox Regression) age >75 and previous history of MI remained significant. With regard to echo parameters, diastolic dysfunction remained as a significant predictor of mortality, whereas LV mass index and LVEF were no longer significant. Detection of Troponin T in the serum was also an independent predictor of mortality during follow up.

**Table 3:** Incidence of adverse events during 2.6 years follow up

**Adverse events**	**Number (%)**
Acute MI	14 (9.1%)
Unstable angina	13 (8.5%)
Cardiac failure	10 (6.5%)
Cerebrovascular accident	6 (3.9%)
Percutaneous coronary intervention	7 (4.6%)
Death from any cause	41 (27%)
**Cause of death:**	
Sepsis	9 (22%)
Renal failure	8 (20%)
Cancer	7 (17%)
Acute MI	4 (10%)
Sudden death	5 (12%)
Cardiac failure	3 (7%)
Cerebrovascular accident	4 (10%)
All cardiovascular death	16 (39%)
Accident	1 (2%)

**Table 4:** Univariate and multivariate predictors of mortality in advanced CKD patients

**Factor**	**Univariate P value**	**Multivariate P Value**	**Hazard ratio**	**Lower 95% CI**	**Upper 95% CI**
Age >75	0.0005	0.002	3.21	1.53	6.74
History of MI	<0.0001	0.007	2.72	1.32	5.64
LVEF < 55%	0.02	NS	NA	NA	NA
High LVMI	0.03	NS	NA	NA	NA
Diastolic grade > 1	0.0005	0.001	3.42	1.66	7.08
Elevated troponin T	<0.0001	0.005	3.70	1.50	9.18

Independent variables entered in the Cox Regression model: age, sex, diabetes, history of MI, LVEF, LVMI, Diastolic grade >1, Troponin T. CI, confidence interval; LVEF, left ventricular ejection fraction; LVMI, left ventricular mass index.

## Discussion

Cardiovascular disease is the leading cause of death in patients with advanced CKD [2]. Our study has shown that patients with advanced CKD had a mortality rate of 27% (39% from cardiovascular causes) over a mean follow up of 2.6 years. The overall mortality rate was higher than in the placebo group in the SHARP Trial which enrolled patients with moderate to severe CKD with one third on dialysis (24.1% after 4.9 years) [15]. This is possibly explained by the higher age of our cohort (66 years v 62 years in SHARP) and the inclusion of more patients on dialysis. The UK Renal Registry of 5447 patients starting dialysis (mean age 64) documented a mortality rate of 29.7% after 3 years [16]. The mortality rate in our non-dialysis group, a cohort which has not been studied extensively in other studies, was surprisingly high at 24%. TREAT which studied diabetic patients with a GFR of 20–60 mL/min/1.73 m^2^ reported a mortality of 20% after a median 2.4 years follow up [17]. The CRIC study which enrolled 3939 patients with stage 2–4 CKD recently reported an overall mortality rate of 9.5% and a combined incidence of MI, CVA and peripheral vascular disease of 8.4% [18]. Mean eGFR in the CRIC study patients was 44.8 ml/min/1.73 m^2^ compared with 22.3 ml/min/1.73 m^2^ in our non-dialysis (stage 4) patients.

The pathophysiology of cardiac disease in CKD is related to the interaction of multiple factors including hypertension, chronic volume overload, anaemia, presence of an AV fistula in patients on dialysis, as well as metabolic factors such as acidosis, hypoxia, hypocalcaemia and high levels of parathyroid hormone [3,4]. Morphological changes in the heart include LVH, advanced coronary atherosclerosis, microvascular disease and diffuse interstitial myocardial fibrosis [19,20]. These abnormalities are common in CKD patients and have been shown in to be predictive of mortality [21,22].

Assessment of diastolic function by echocardiography has shown a high incidence of abnormalities in dialysis and non-dialysis CKD patients [23,24]. Some investigators have found abnormalities in tissue Doppler velocity in virtually all patients with CKD, suggesting a degree of subclinical myocardial disease in all such patients [25]. In the CRIC study (stage 2–4 CKD) diastolic function was abnormal in 71% of patients [26]. In our cohort some degree of diastolic dysfunction was present in 85% of patients and 35% had grade 2 or higher diastolic dysfunction, which was a powerful independent predictor of mortality. Patients with abnormal diastolic function have been found to have increased integrated backscatter which is a measure of collagen content of myocardial tissue [27]. We used E/é as part of our grading of diastolic function and this has been shown to reflect LV filling pressure and has been associated with mortality in CKD patients [24].

We detected troponin T in 38% of non-dialysis and 79% of dialysis patients. Troponin T was detected in 45% of diabetic CKD patients in TREAT and was an independent predictor of adverse outcomes in this and other studies [17,28]. The precise mechanism for increased Troponin T concentrations in advanced CKD patients is not clear. Acute myocardial ischaemia may be a factor in a minority of patients as suggested by an MRI study in ESRD patients with raised troponins [29]. None of our patients had clinical evidence of myocardial infarction at the time of their blood test. A post-mortem study of patients with raised troponin T which included 6 haemodialysis patients found histologic evidence of myocardial infarction in some patients but also other cellular pathologies in virtually all subjects including degenerative changes associated with heart failure, inflammation, fibrosis, endocarditis, sepsis, amyloid deposition, and infiltration by tumour [30]. The concept of myocyte damage in these patients is supported by findings of a higher incidence of LV dilatation, increased LV mass, impaired systolic function and raised LV filling pressures [24]. The situation is made more complex by availability of high sensitivity assays which detect troponin T in virtually all advanced CKD patients [31]. In the CRIC study high sensitivity Troponin T was detected in 84% of stage 2–4 CKD patients and was associated with LVH and systolic dysfunction [32].

Detection of significant diastolic dysfunction and elevated troponin T can be early markers of myocardial disease and increased risk in advanced CKD. However, the optimal management of these patients is not well defined. Therapeutic modalities that have been successfully used include aggressive lipid lowering [15], treatment for hypertension and anaemia [33], increased frequency of dialysis [34], implantable defibrillator insertion [35], coronary revascularisation [36], multiple risk factor intervention [37] and renal transplantation. The application of these therapies should be considered after review of treatment guidelines and careful clinical assessment of individual patients.

### Limitations

This was a single centre study and our patient population was relatively small, raising the possibility of selection bias. However, subjects were enrolled consecutively and followed closely for a median period of almost 3 years. As in all observational studies, no conclusions can be drawn regarding causality, even though the associations between diastolic function, serum troponin and mortality were strong and independent of age, sex, and other factors adjusted for in the multivariate model. Our study included a combination of dialysis and non-dialysis patients and this may make the results more difficult to interpret, although we felt the inclusion and comparison of the two groups was valuable.

## Conclusion

Patients with advanced CKD have a high incidence of structural cardiac abnormalities including increased LV mass, diastolic dysfunction and raised troponin T. These factors are associated with increased mortality and adverse cardiac events compared to CKD patients without these factors, even in the non-dialysis population. We therefore suggest routine evaluation of diastolic function by echocardiography, measurement of serum troponin T and screening for vascular risk factors in all CKD patients. Whether early identification of these risk markers and intervention in CKD patients will lead to improved outcomes should be the subject of further investigation.

## Competing interests

The authors declare that they have no competing interests.

## Authors’ contributions

AF conceived and designed the study, analysed the results and drafted the manuscript. RP participated in drafting the manuscript and analysing the results. GT participated in designing the study and writing the manuscript. BS participated in designing the study and performed the statistical analysis. LA participated in data analysis and drafting the manuscript. All authors read and approved the final manuscript.

## Pre-publication history

The pre-publication history for this paper can be accessed here:

http://www.biomedcentral.com/1471-2369/14/280/prepub
